# Atomic-Resolution EDX, HAADF, and EELS Study of GaAs_*1-x*_Bi_*x*_ Alloys

**DOI:** 10.1186/s11671-020-03349-2

**Published:** 2020-05-25

**Authors:** Tadas Paulauskas, Vaidas Pačebutas, Renata Butkutė, Bronislovas Čechavičius, Arnas Naujokaitis, Mindaugas Kamarauskas, Martynas Skapas, Jan Devenson, Mária Čaplovičová, Viliam Vretenár, Xiaoyan Li, Mathieu Kociak, Arūnas Krotkus

**Affiliations:** 1 Center for Physical Sciences and Technology, Saulėtekio al. 3, Vilnius, Lithuania; 2 STU Centre for Nanodiagnostics, University Science Park Bratislava Centre, Slovak University of Technology, Vazovova 5, Bratislava, Slovakia; 3 Solid State Physics Laboratory, University of Paris SUD, 91400 Orsay, France

**Keywords:** GaAsBi, Dilute bismides, Atomic resolution HAADF, HAADF image quantification, Bulk plasmon mapping, Atomic resolution EDX, Monochromated EELS

## Abstract

The distribution of alloyed atoms in semiconductors often deviates from a random distribution which can have significant effects on the properties of the materials. In this study, scanning transmission electron microscopy techniques are employed to analyze the distribution of Bi in several distinctly MBE grown GaAs_*1−x*_Bi_*x*_ alloys. Statistical quantification of atomic-resolution HAADF images, as well as numerical simulations, are employed to interpret the contrast from Bi-containing columns at atomically abrupt (001) GaAs-GaAsBi interface and the onset of CuPt-type ordering. Using monochromated EELS mapping, bulk plasmon energy red-shifts are examined in a sample exhibiting phase-separated domains. This suggests a simple method to investigate local GaAsBi unit-cell volume expansions and to complement standard X-ray-based lattice-strain measurements. Also, a single-variant CuPt-ordered GaAsBi sample grown on an offcut substrate is characterized with atomic scale compositional EDX mappings, and the order parameter is estimated. Finally, a GaAsBi alloy with a vertical Bi composition modulation is synthesized using a low substrate rotation rate. Atomically, resolved EDX and HAADF imaging shows that the usual CuPt-type ordering is further modulated along the [001] growth axis with a period of three lattice constants. These distinct GaAsBi samples exemplify the variety of Bi distributions that can be achieved in this alloy, shedding light on the incorporation mechanisms of Bi atoms and ways to further develop Bi-containing III-V semiconductors.

## Introduction

The bismide GaAs_*1−x*_Bi_*x*_ alloy has experienced an extensive amount of research and represents the emerging class of bismuth-containing group III–V semiconductors [1]. Bismuth is the largest stable and non-toxic element, which upon incorporation produces large GaAs band gap reduction. Substitution of Bi in the group V sublattice allows achieving the band gap bowing as much as 90 meV/Bi% in GaAsBi with a moderate lattice strain [1–3]. A large spin-orbit band splitting is another notable effect of Bi incorporation in the lattice. This may allow suppressing the inter-valence band absorption and Auger-Meitner recombination in GaAs_*1−x*_Bi_*x*_ with concentrations *x* > 10% [4]. Combined with the reduced band gap sensitivity, these properties make the bismide an attractive candidate for applications in the long-wave infrared lasers, photodetectors, and multijunction solar cells, among others [1, 5–7].

The incorporation of Bi in the GaAs matrix requires unconventional growth conditions since Bi atoms tend to desorb at typical GaAs growth temperatures [8–10]. Substrate temperatures below 400 °C are typically needed, as well as nearly stoichiometric group III/V ratios. Care must be taken to avoid the formation of surface Ga or Bi droplets which can easily occur near these conditions and are associated with non-uniformities of the growing layer thickness and its composition [11–14]. Low temperatures needed to incorporate larger concentrations of Bi make molecular-beam epitaxy (MBE) the preferred method of synthesis, although progress has been made using metal-organic vapour phase-epitaxy [15–17]. GaAsBi alloys still show surprisingly high photoluminescence (PL) intensity for these low growth temperatures, which is attributed to the Bi surfactant effect and reduced density of As-related point defects that typically form in low-temperature GaAs [18, 19]. In the picture of the valence band (VB) anti-crossing, incorporated individual Bi atoms produce a resonant state below the extended GaAs VB causing the optical band gap reduction [2, 20, 21]. First-principles calculations also demonstrate that clusters composed of nearby interacting Bi atoms can produce band gap narrowing significantly larger than by isolated Bi atoms [22]. These different Bi configurations produce strong VB perturbations and can introduce localized electronic defect states. Studies suggest that lattice strain produced by large Bi atoms will cause the clusters more likely to bind to vacancies V_Ga_ and V_As_ [23]. As-rich growth conditions should favor the formation of Bi_Ga_ hetero-antisite defects which are predicted to cause deep hole traps in GaAsBi [23, 24]. Pronounced exciton localization effects are commonly observed in temperature-dependent PL of GaAsBi alloys and are attributed to such Bi-related clusters and defect complexes [25, 26].

Like many other ternary III–V semiconductor alloys, GaAsBi shows the tendency for spontaneous ordering [27]. The so-called CuPt_B_-type ordering, whereby the concentration of Bi atoms is modulated on every second {111}B-type plane, has been observed using high-resolution (scanning) transmission electron microscopy (STEM/TEM) [13, 17, 28]. It is widely accepted that the CuPt_B_-type modulation in III–V alloys is driven by surface reconstruction dynamics and is accompanied by the (2 × 1) reconstruction consisting of surface dimer rows [27, 29–33]. When deposited on flat (001) GaAs substrates, the ordering occurs on two of the four distinct sets of {111} planes. A single B-type ordering subvariant can be further selected by utilizing vicinal substrates. Indeed, recent work showed this to apply for GaAsBi as well, whereby large CuPt_B_-type domains have been achieved on a single set of {111}B planes using low-angle offcut wafers [34]. The CuPt-type ordering in GaInP_2_ is probably the most studied since high-quality crystals with large order parameter can be achieved in this alloy. The long-range order changes the zinc-blende point group symmetry from tetrahedral T_d_ to trigonal C_3v_ [35, 36]_._ Notable effects due to the symmetry reduction include band gap narrowing, the polarization of photoluminescence, birefringence, and anisotropic strain [37–39]. The magnitude of these effects depends on the long-range order parameter, *η*, which shows the extent of elemental distribution among ordered lattice planes. In a CuPt_B_-ordered AB_*1−x*_C_*x*_ alloy (for *x* < =0.5), the lattice alternates in B element-rich AB_1−(*x−η*/2)_C_*x−η*/2_ and C-rich AB_1-(*x + η*/2)_C_*x + η*/2_ monolayers along a < 111>B direction. The order parameter *η* = 0 in a random alloy while in a fully ordered one with concentration *x* it is thus *η* = 2*x*.

Clearly, the distribution of Bi within such ordered alloys differs from a random alloy, and this should be considered when further deducing the alloy properties [17, 40]. The understanding of CuPt-ordering effects in dilute GaAsBi alloys is still at the early stages, requiring more systematic studies. In this article, advanced aberration-corrected STEM methods are employed to analyze modes of Bi distribution in several distinctly grown GaAsBi alloys. The analysis is performed using statistical STEM Z-contrast image processing and image simulations, as well as atomically resolved X-ray energy dispersive spectroscopy (EDX). Monochromated electron energy-loss spectroscopy (EELS) is employed to investigate local unit-cell volume changes in GaAsBi using bulk plasmon energy shifts.

## Results and discussion

**Fig. 1:**
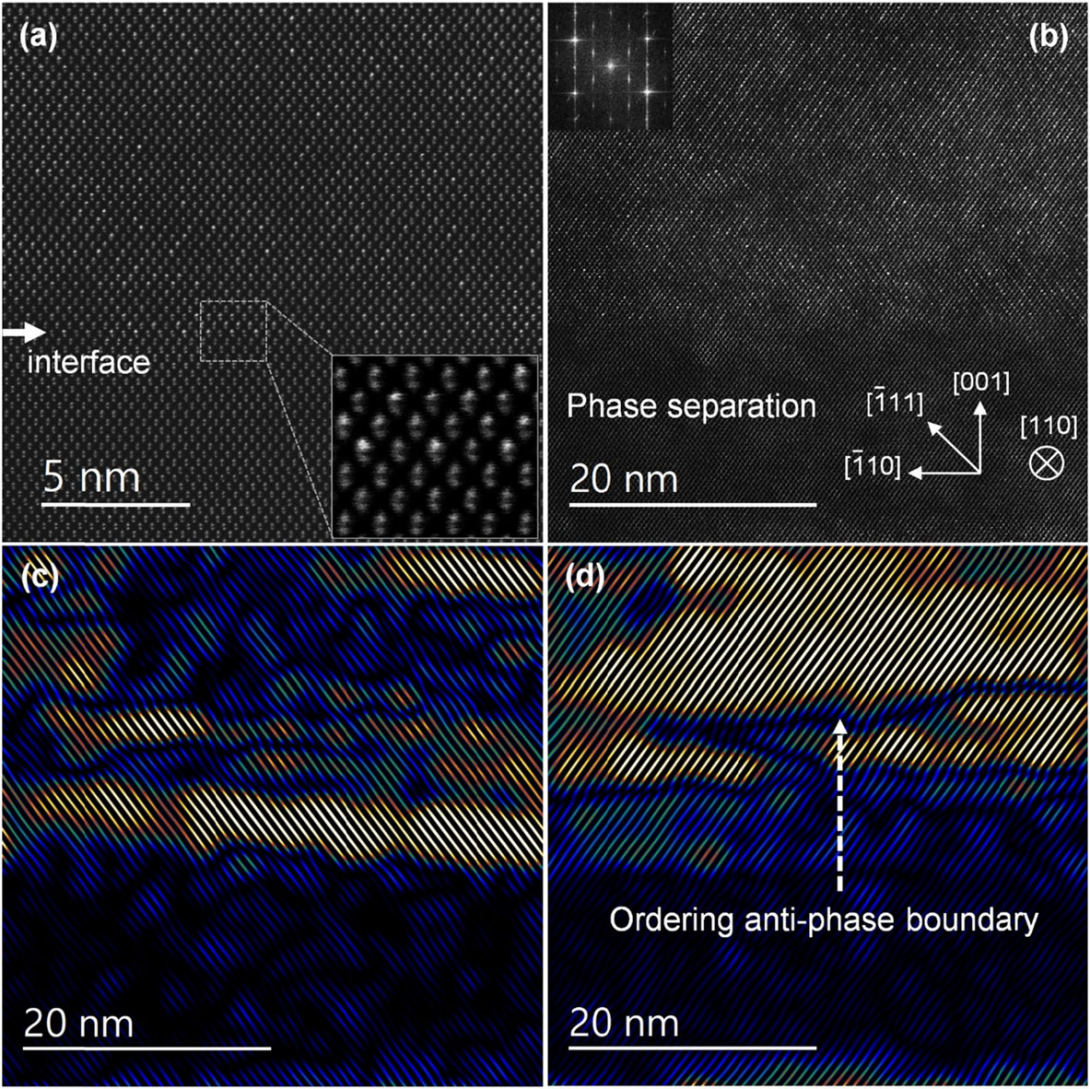
**a** Cross-sectional HAADF image of sample S1 GaAs-GaAsBi interface area. A zoomed-in inset of the interface is shown on the bottom right. Crystallographic directions are the same as in Fig. 1b. **b** HAADF image of the sample away from the interface. An elongated spontaneously phase-separated GaAs-like domain is seen in the middle. Inset shows the Fourier transform of the image. **c** An image formed from (**b**) using the 1/2[$\overline{1}$11]* pair of superlattice reflections. Brighter colors indicate more pronounced ordering. **d** An image formed from (**b**) using the pair of ½[1$\overline{1}$1]*reflections

The first GaAs_*1−x*_Bi_*x*_ sample presented here, S1, is a p-i-n hetero-diode with doped GaAs layers and intrinsic 420 nm bismide. Bismuth concentration in the sample was determined to be 4.5% Bi using X-ray diffraction (not shown here) and room temperature PL, indicating 1.10 eV band gap (SI Fig. S1). PL band edge measurements are translated to Bi% using references [1, 2, 4]. A cross-sectional atomic-resolution HAADF STEM image along [110] zone axis near the GaAs-GaAsBi interface is shown in Fig. 1a. The [001] growth axis and other relevant crystallographic directions are marked in Fig. 1b and also applies to Fig. 1a. Since heavy Bi atoms scatter probe electrons to high angles much stronger than Ga or As atoms, HAADF detector with a large inner-collection angle (90 mrad here) favorably highlights Bi distribution in thin samples. The crystal viewed along a <110> direction appears as a collection of atomic “dumbbells”, which are oriented parallel to the growth [001] axis. Due to their similar atomic numbers (Z), Ga (31) and As (33) cannot be easily distinguished by mere inspection of the HAADF images. However, Bi-containing group-V columns show noticeably higher contrast. As can be seen in Fig. 1a and in the zoomed-in region near the interface, group-V columns are positioned in the top half of dumbbells above Ga columns. This is expected when imaging GaAsBi along [110] zone axis. Note that the polarity of group V/III dumbbells is reversed when the sample is viewed along the orthogonal [$\overline{1}$10] direction. These in-plane orthogonal directions can also be distinguished since the CuPt-type ordering occurs on {111}B type planes and thus can only be seen by imaging along [110] zone axis. Figure 1b shows a lower magnification HAADF image deeper within the film with pronounced CuPt_B_-type ordering. The ordered domains are seen to alternate randomly between the two sets of {111}B planes, i.e., ($\overline{1}$11) and (1$\overline{1}$1). These are termed B_+_ and B_−_ subvariants by convention. Fourier transform of the image is shown in the top left inset. The four main Bragg spots are [111]*-type, while the four 1/2[111]*-type superlattice spots indicate CuPt_B_ ordering with similar magnitude on the two sets of {111}B planes. A phase-separated GaAsBi region is visible in Fig. 1b as a darker stripe in the lower image portion. This domain appears darker than a bismide since it is Bi-deficient GaAs-like. Due to the metastability of GaAsBi alloys, a spinodal decomposition and phase separation have been reported in many articles [11–14, 41, 42]. For a clearer depiction of the B_+_ and B_−_ subvariant ordering, Figs. 1c, d are presented by forming images using 1/2[111]* superlattice reflection pairs. A mask is applied to each superlattice pair in the reciprocal space and inverse Fourier transformed back to the real space. Brighter regions of (111) planes in these images indicate that the ordering is more pronounced, or in other words, that the order parameter is varying locally. There are also TEM sample surface thickness variations due to the sample preparation by a focused ion beam. The sample preparation can leave amorphous surface layers and melted Ga atomic agglomerates on the surface, which can cause weak image intensity modulations. However, Ga scatters much weaker to the high angles than Bi atoms and should not significantly influence the analysis of Bi distribution. The arrow in Fig. 1d shows a region with ordering anti-phase boundaries. Across such a boundary, the B_+_ (B_−_) domain changes its phase by switching all Bi-rich planes into As-rich planes. Ordering anti-phase boundaries can form by glide of dislocations or due to a random alternation among B_+_ and B_−_ domains during growth [43]. The latter appears to be the case here.

**Fig. 2:**
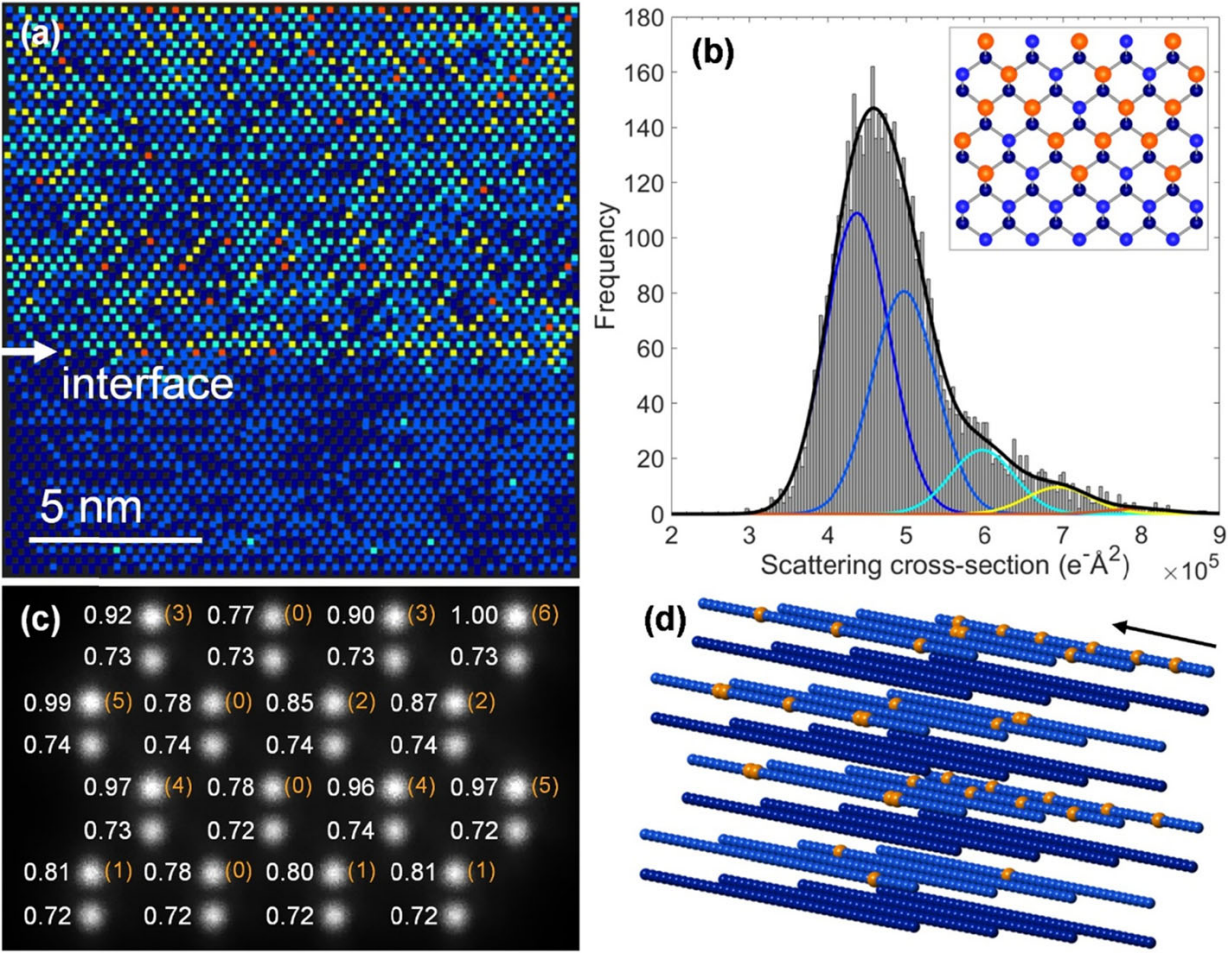
**a** A distribution of SCS in Fig. 1a. The colored squares on each atomic column are according to the SCS color scheme in Fig. 2b. **b** A histogram of SCS in Fig. 1a, fitted with 5 Gaussians. The inset shows a depiction of the interface region. Ga atomic columns are in dark blue, As—in lighter blue, and columns containing Bi are in orange. **c** Simulated HAADF image of the GaAsBi structure shown in Fig. 2d. The number of Bi atoms in a column is shown in parentheses in orange to the right of each group-V column. The fitted SCS values are shown to the left of each column and are normalized to the largest SCS value in the supercell. **d** The model GaAsBi <110> structure rotated sideways to highlight Bi positions (orange), light blue-As, dark blue-Ga atoms. The arrow shows the incident beam direction

Quantification of the HAADF image shown in Fig. 1a is carried out next by considering spatial distributions of atomic column scattering cross-sections (SCS) (see Methods). The StatSTEM algorithm is used to fit columns with 2-dimensional Gaussians, and the SCS of a given column is defined as volume under that Gaussian [44, 45]. This a parametric model-based quantification, as opposed to direct integration of column intensities in an experimental image. The parametric model approach can be more reliable if column intensities tend to overlap, such as in <110> GaAsBi. The distribution of SCS in the quantified Fig. 1a is plotted as a histogram in Fig. 2b, which is tentatively fitted with five Gaussians. The spatial distributions of SCS are then plotted in Fig. 2a on the model structure composed of a superposition of Gaussians using the same color-scheme squares placed on each column. The SCS of Ga and As columns strongly overlap and produce the main peak in the histogram. This is due to similar Z-numbers of these atoms as well as additional experimentally introduced broadening (see Methods). By plotting the lower Gaussian component (dark blue color) or the upper (lighter blue) within this main peak shows that ~ 60% of, e.g., As columns in the GaAs buffer layer are identified correctly, as can be inspected from the dumbbell polarity. For comparison, SCS quantification of the bottom GaAs buffer layer alone is presented in Supplementary Fig. S2. It suggests that more than two Gaussians are needed presently to better distinguish Ga and As columns in the field of view and indicates that their mean SCS differs by as much as 10%. This difference agrees with our simulations shown below and also the results found in Beyer et al. [17], where Ga and As integrated column intensity distributions in [010] GaAsBi were resolved. The presence of strongly scattering Bi atoms extends the SCS to values above ~ 5.5 × 10^5^ e^-^Å^2^ (see SI Fig. S2), which gives rise to the right-hand shoulder in Fig. 2b. It is fitted with three Gaussians to tentatively distinguish columns with higher Bi content. Atomically abrupt GaAs-GaAsBi interface can be seen in Fig. 2a. Closer inspection shows that the first group-V (001) layer of columns containing significant number of Bi atoms is arranged on every second dumbbell along the interface. This suggests the onset of CuPt-type ordering early in the epitaxial growth. A depiction of the interface atomic configuration is shown in the inset of Fig. 2b. It recreates the arrangement of atoms along the interface with Bi atoms (orange) on every second column along the first group-V (001) plane. The first ~ 4–5 (001) atomic planes in Fig. 2a show no predisposition for CuPt B_+_ or B_−_ subvariants. More pronounced single-variant ordering emerges from the ~ 6th (001) group-V atomic layer and then switches to the other subvariant. No antisite defects Bi_Ga_ are indicated by the SCS distribution at the interface, which would be visible as squares on group-III columns with colors associated with Bi. A likelihood of several Bi_Ga_ antisites, however, is found in the top right corner of the figure. Both, group-III and group-V columns show Bi-like SCS on single dumbbells in that region, which may also indicate the presence of defect pairs Bi_Ga_-Bi_As_. To get a better idea of the number of Bi atoms involved in determining the SCS, note that in a nominally 20–25-nm thick sample there are 50–60 atoms in a < 110> column. Thus, 2–3 Bi atoms are most likely to be found in a group-V column for a random alloy with presently 4.5% Bi. This number will be higher in ordered Bi-rich planes, probably reaching up to 6–7 in columns with the largest SCS [40]. To supplement the StatSTEM analysis, multislice HAADF image simulation is presented next on a model GaAsBi <110> supercell structure (see Methods for details).

The contribution to HAADF image intensity from Bi atoms at different sample depths can be non-linear due to what is loosely termed as channeling [46–49]. Quantification of dopants at the atomic scale hence requires consideration when distinguishing true variation in composition from the variation in dopant configurations [50, 51]. To illustrate the channeling behavior, the average probe intensity variation with sample depth when positioned over As column in <110> GaAs is numerically simulated and shown in Supplementary Fig. S3 (see Methods). The model GaAsBi structure 17 nm thick used for HAADF simulations is shown in Fig. 2d rotated sideways to highlight positions of Bi atoms (orange) within As columns (As—light blue, Ga—dark blue). The arrow marks the incident beam direction. The simulated image shown in Fig. 2c is fitted using the StatSTEM algorithm for comparison to the experiment. The obtained SCS values were normalized to the SCS value of the column with the largest SCS (6 Bi atoms) and rounded to two significant digits. These normalized values are shown to the left of each column. The number of Bi atoms in each group-V column is shown in parentheses to the right of the column. In a reasonable agreement with the previous findings, the difference between As and Ga SCS values is found ~ 8%. The difference in SCS between pure As column and As columns containing one Bi atom is in a range 2–4% for differently positioned Bi atoms. One can clearly see that different Bi configurations can be misinterpreted for different compositions, e.g., 4 and 5 atoms or 5 and 6 atoms, which give nearly the same SCS values. Bi atoms in a column towards the bottom surface contribute increasingly less to the SCS. Several configurations having 2 Bi atoms one after another examined here seem to produce large contributions to the SCS values. A configuration of 2 Bi atoms along a [110] column can be expected to be found in practice if the CuPt_B_ ordering in GaAsBi alloys indeed produces structural units with C_3v_ point group symmetry, i.e., a Ga atom with nearest neighbours 1 As and 3 Bi atoms. Note that identical Ga columns also show variation in their SCS by up to ~ 0.02. This suggests that their immediate environment, e.g., nearby strongly scattering columns, contributes additional intensity due to multiple scattering or by coupling to them via extended probe tails [52]. Recently introduced better scaling algorithms open the possibility to speed up the quantum mechanical multislice computations and thus to explore aforementioned effects in more detail [53, 54].

To conclude the STEM analysis of sample S1, electron energy-loss spectroscopy (EELS) is used to map the bulk plasmon energies. The plasmon energy shifts will be related to the unit-cell volume changes and thus to the alloy strain, as discussed next. GaAs exhibits one major plasmon peak at ~ 16 eV, and unlike, e.g., CdTe, it does not show complex interfering features from inter-band transitions [55]. As a first approximation to interpret the measured plasmon energy changes, we employ the Drude-Lorenz model for free-electron electron gas, where free electrons are now the valence electrons in the semiconductor [56]. The bulk plasmon energy in this model is given as ${E}_p=\hslash {\left(N{e}^2/ Vm{\epsilon}_0\right)}^{1/2}$, where *N* is the number of valence electrons in the unit cell, *e* is the electron charge, *V* is the unit-cell volume, *m* is the electron mass, and *ε*_*0*_ is the permittivity of free-space. The simple Drude-Lorenz model generally predicts the plasmon energy within a few percent in semiconductors and needs to be corrected for band structure effects if a better match is sought after [56]. As shown in InGaAs and group-III nitride semiconductor alloys, the change in unit-cell volume is the leading quantity determining the plasmon energy shifts [57, 58]. Similarly, the substitution of isoelectronic Bi atoms in GaAs matrix mainly acts to expand the unit-cell volume, *V*, and thus red-shift the plasmon energy. In the following, we employ the measured GaAs and GaAsBi peak energies to infer the local strain-state change in GaAsBi layer via a ratio of their unit-cell volumes.

**Fig. 3:**
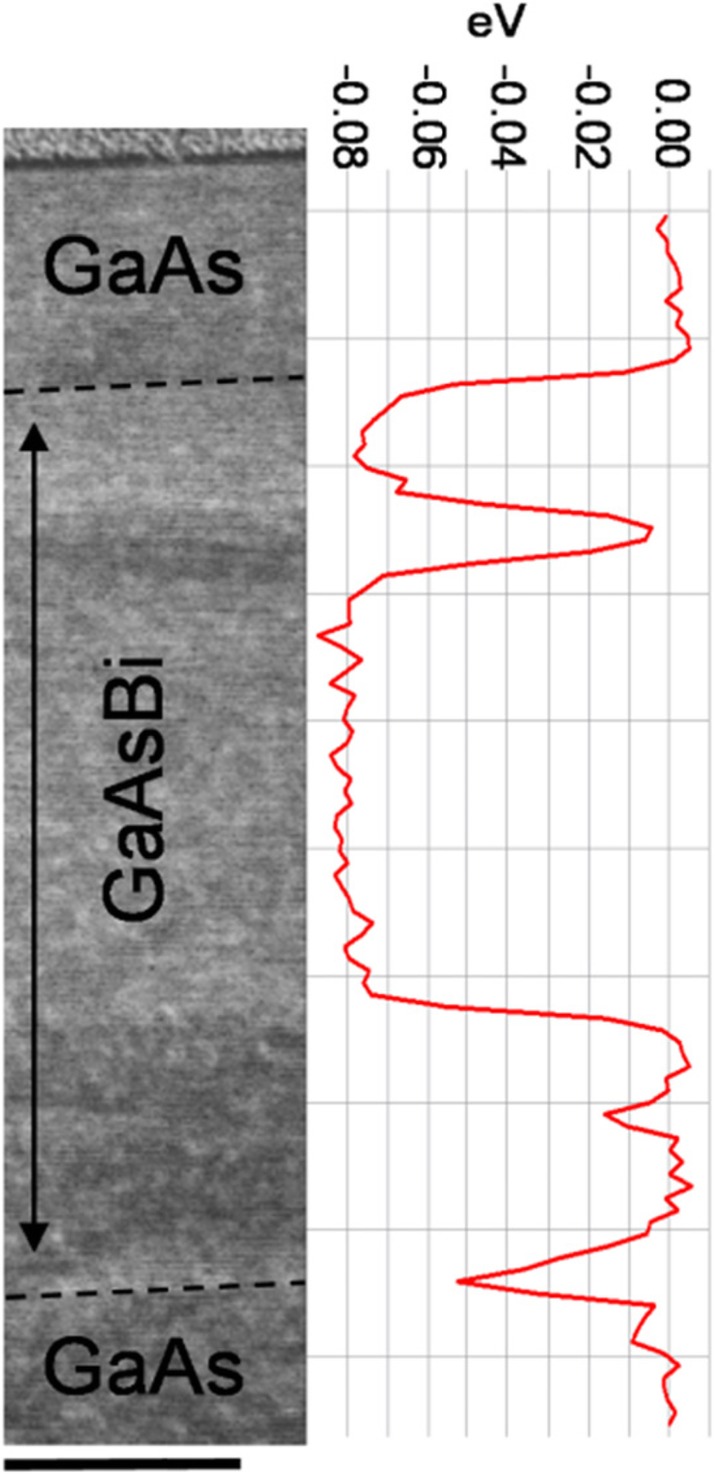
Cross-sectional HAADF image (left) of the p-i-n sample S1, with marked GaAs and GaAsBi layers. Darker regions within GaAsBi are phase-separated domains. The line profile (right) shows EELS bulk plasmon peak energy shift, *E*_GaAsBi_-*E*_GaAs_, relative to the GaAs buffer layer. The profile is closely aligned with the simultaneously acquired HAADF image on the left. EELS data pixels are fully binned in the horizontal direction and thus indicate spatially averaged values. The scale bar is 100 nm, and it also applies to the vertical axis of the EELS profile

A region is selected which contains phase separated GaAsBi domains shown in the HAADF image Fig. 3. EELS spectra were collected from each pixel accompanying the simultaneously acquired HAADF image (see Methods and SI Fig. S4 for raw spectrum). Dashed lines in the HAADF image indicate the interfaces between the intrinsic GaAsBi and the p-type (bottom) and n-type (top) GaAs layers. The interface demarcation lines were determined from lower magnification STEM images (not shown here). The protective Pt layer is visible as the higher contrast material above the upper n-GaAs. GaAs layers and also phase separated domains within GaAsBi appear darker in the HAADF image. The vertical line profile on the right-hand side EELS figure was acquired by binning all EELS data points horizontally. It shows relative bulk plasmon peak energy shift, E_GaAsBi_-E_GaAs_, as referenced to the GaAs plasmon energy (measured to be 16.23 eV) within the bottom p-GaAs buffer layer. The plasmon peak is seen to shift on average by 0.08 eV to lower energies in the GaAsBi layer. The small variations within ~ 0.01 eV are at the quantification noise levels. The phase separated domains near the top GaAs (thin layer) and bottom (two intersecting domains) return to the GaAs plasmon energy value, suggesting that they contain negligible Bi concentrations. Dopant concentrations in the GaAs layers (order 10^17^ cm^−3^) are insignificant compared to *N*/*V* and should not affect the plasmon energy. We now consider two limiting cases for the GaAs_*1−x*_Bi_*x*_ unit-cell volume *V*; one where the lattice is fully relaxed and another where it is fully strained to GaAs substrate. In the fully relaxed case, the unit cell is cubic with lattice constant *a* ≈ 5.684 Å at *x* = 4.5% Bi [1]. Using the above square root relationship between plasmon energy and *V*, the energy shift relative to GaAs should be $\Delta {E}_p^{GaAs Bi}=16.23\left({\left({V}_{GaAs}/{V}_{GaAs Bi}\right)}^{1/2}-1\right)=-0.132\mathrm{eV}$, which is clearly larger than the measured one. Based on relaxation trends of GaAsBi alloys, we estimate that ~ 30% of the lattice is relaxed in this 420 nm thick film, considering that it also experienced short thermal annealing while growing the top n-GaAs layer. Hence, the average GaAsBi unit cell will be overestimated in the fully relaxed scenario and explains larger $\Delta  {E}_p^{GaAsBi}$ obtained above. In the other limit, the bismide lattice is taken to be fully strained with the in-plane lattice constant equal to that of GaAs (*a* = 5.653 Å). The out-of-plane lattice constant required to retrieve the − 0.080 eV energy shift is then found *a*_z_ = 5.709 Å. This is a sensible *a*_z_ value and can be compared to the XRD-RSM measurements of GaAsBi compressively strained to GaAs substrate [1, 34, 59]. Due to relaxation, the actual lattice constants are expected to be in between these two limiting cases. This demonstrates a promising characterization method which can provide information on the lattice strain complementary to X-ray-based techniques in such metastable alloys.

**Fig. 4:**
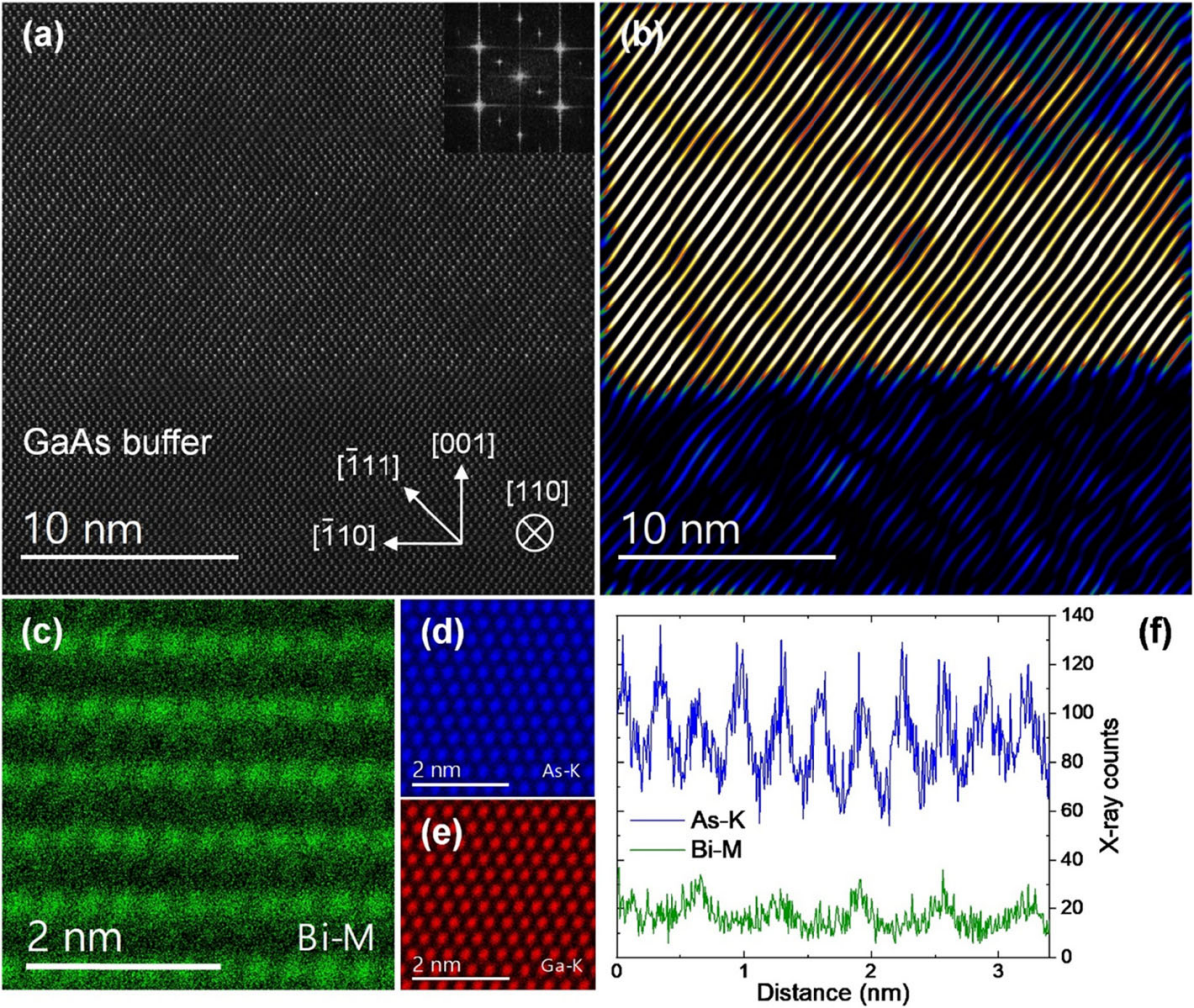
**a** HAADF image of GaAsBi sample S2 near the interface with the GaAs buffer layer, grown on offcut (001) Ge. Fourier transform on the top right shows pair of peaks indicating ordering on the single set of ($\overline{1}$11) planes. **b** An image formed from (**a**) using the pair of superlattice 1/2[$\overline{1}$11]* Bragg spots. **c**–**e** Wiener filtered EDX images of the GaAsBi sample, with Bi-M, As-K, and Ga-K X-ray emissions as indicated. Note that in EDX data, the crystallographic directions are rotated to align the ordered ($\overline{1}$11) planes horizontally. **f** Horizontally summed vertical X-ray counts profile of the raw As-K and Bi-M signal. Two aligned data sets are combined to obtain the profile

The second GaAsBi sample, S2, was grown over a GaAs buffer layer that was deposited on an offcut Ge substrate (see Methods). The Ge-GaAsBi hetero-epitaxy was analyzed in our previous work, which also demonstrated large-domain single-variant CuPt_B_ ordering in GaAsBi [34]. Additional data are presented in this work and are used for completeness of the discussion on Bi atomic ordering. The total bismuth concentration in this sample is ~ 5.8% as measured by PL (SI Fig. S1) [34]. The offcut combined with GaAs buffer layer employed in this epitaxy helps to avoid the formation of anti-phase domains in GaAsBi, which are still troublesome to eliminate when growing it directly on non-polar Ge [60–62]. Figure 4a shows the GaAs-GaAsBi interface area with GaAsBi layer visibly brighter in the HAADF image. As opposed to the previous GaAsBi film deposited on a flat GaAs substrate, here, a single CuPt_B_ ordering subvariant is selected due to the offcut. This can be seen in the HAADF image, and its Fourier transform inset on the top right showing a pair of 1/2[$\overline{1}$11]* superlattice spots. Figure 4b was formed by applying a mask to the pair of superlattice reflections, analogous to Fig. 1c, d. It shows a much more uniform and large-domain ordering in the film. Atomically resolved EDX images were acquired from this sample to estimate the order parameter *η* based on the compositional analysis. EDX chemical mapping often excels over the alternative core-loss EELS quantification, which tends to have worse signal-to-noise ratio when quantifying high-energy and delayed ionization edges [56, 63–65]. STEM scanning direction was changed to align the ordered ($\overline{1}$11) planes horizontally. Figure 4c–e shows the Wiener filtered X-ray elemental maps. The ordering of Bi atoms on every second ($\overline{1}$11) plane is clear and it follows As atomic positions. For the EDX compositional quantification, two data sets with 512 × 512 pixels each were acquired from different areas of the sample using identical experimental conditions. Subregions were aligned, and the raw signals summed resulting in a total of 10 frames. A horizontally summed raw data vertical line profiles of As-K and Bi-M are shown in Fig. 4f. To quantify bismuth composition in the Bi-rich and Bi-deficient (111) planes, an integration window 3 Å wide was used, centered on the atomic planes. After background subtraction and averaging over all (111) planes, it shows that Bi X-ray counts are ~ 3 times higher in the Bi-rich planes. Total concentration 5.8% Bi in the sample, as obtained by PL and XRD-RSM measurements, is then used to linearly scale Bi X-ray counts to the composition, which shows that Bi reaches up to 9% in the Bi-rich planes. The order parameter can thus be estimated (see Introduction) to be *η* = 0.07. Note that a fully ordered bismide with this total Bi concentration would have the order parameter η = 0.116. Similarly to HAADF analysis, EDX quantification of individual columns suffers from channeling effects since the ionization potential of core electrons is highly localized. As shown by other authors in Al_*x*_Ga_*1-x*_As alloy, this can result in up to ~ 5% X-ray counts standard deviation due to different dopant configurations [50]. Considering the deviation, the X-ray counts are still found to scale linearly with a number of dopants in not too thick samples. The configurational error in the present study is minimized by effectively averaging over ~ 11 atomic columns in each (111) plane, for a total of ~ 130 columns. In addition, electron probe tails and multiple scattering can produce signal delocalization in EDX images [52]. HAADF image simulations in the previous section showing variation of the Ga SCS values hint on the expected magnitude of these effects in the EDX quantification. The shot noise appears presently to be the main accuracy limiting factor due to inherently low Bi X-ray counts in such dilute alloys.

**Fig. 5:**
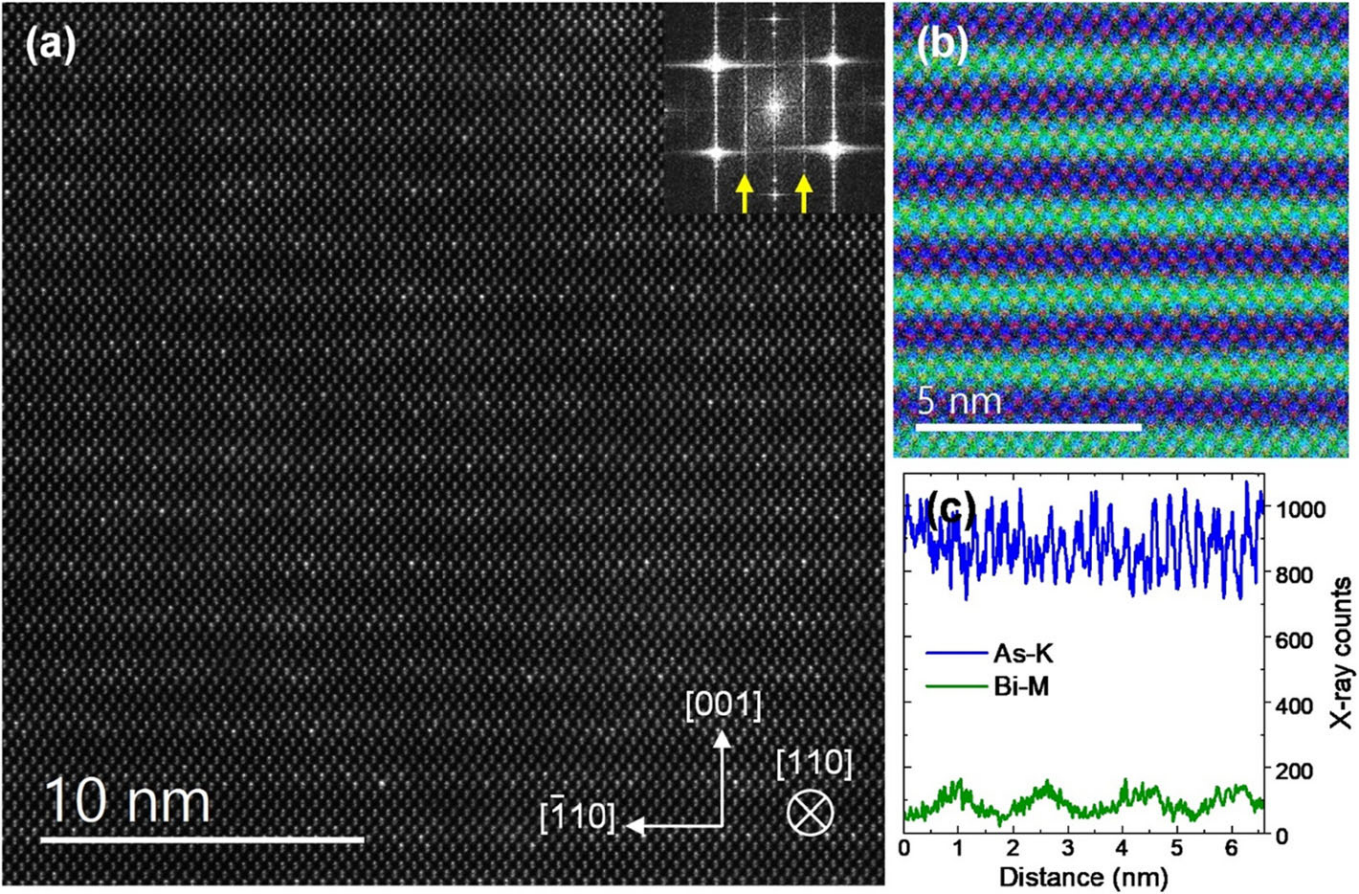
**a** HAADF image of the VCM GaAsBi sample S3. Bi concentration modulations along the growth [001] direction are visible, as well as CuPt_B_ ordering within the Bi-enriched planes. Inset shows Fourier transform with reminiscent CuPt_B_ ordering modulated by the VCM. **b** A combined Wiener filtered elemental EDX image of the sample with normalized X-ray counts for each element, Bi-green, As-Blue, and Ga-red. **c** Vertical X-ray count profile extracted from 3 × 3 binned raw As-K and Bi-M signals, horizontally summed within a 5 nm window

The final GaAsBi sample, S3, that we wish to explore here was synthesized under conditions to create so-called vertical composition modulations (VCM) (see Methods) [66]. In contrast to samples S1 and S2, the VCM is achieved in S3 by utilizing a slower substrate rotation rate (RPM), which is coupled to intrinsically inhomogeneous elemental flux profiles reaching the substrate in a typical MBE chamber. The III/V elemental ratio within a sample region can be oscillated by controlling the RPM and the film growth rate to obtain the desired VCM period. A vertical spiral in regards to Bi concentration can be obtained in GaAsBi this way, as has been well explained in *M.A.Stevens* et al. [66]. A cross-sectional HAADF image of the GaAsBi sample S3 is shown in Fig. 5a, grown on a (001) GaAs substrate rotated 5 RPM and 300 nm/h growth rate. Total bismuth composition in the sample area under investigation was determined to be 2.8% Bi using room-temperature PL (SI Fig. S1). The VCM is visible with a well-defined superlattice-like appearance. The tendency to CuPt_B_ order is also visible in this image, and here it incurs the additional vertical modulation. The inset on the top right shows Fourier transform of the image with arrows marking the pair of stripes, which result from Bi content modulation along the [$\overline{1}$10] direction on every second plane and accordingly reduced extent of (111)-type ordered planes along [001].

The sample was also investigated using atomic scale elemental EDX mapping. Figure 5b shows overlaid normalized and color-coded X-ray signals of Bi-M (green), As-K (blue), and Ga-K (red). The corresponding elemental Wiener filtered maps are shown in SI Fig. S5. The peak-to-peak distance between Bi-rich regions is 1.7 nm, which indicates the VCM period is ~ 3 lattice constants. The peak positions do not align on a single Bi-rich (001) plane. This offset reflects that Bi atoms with higher concentrations are dispersed over 2–3 group-V (001) atomic planes, which is clearer in the HAADF image (Fig. 5a). Figure 5c shows 4 VCM periods by plotting vertical Bi-M and As-K line profiles of horizontally summed counts in a 5 nm wide window from the 3 × 3 binned raw EDX data. Despite the signal noise, As-K X-ray count profile seems to inversely follow the Bi-M profile showing small dips at Bi-enriched regions. Such a correlation between substitutional element and the host element X-ray signals may be exploited in future atomic scale EDX analysis of dilute alloys.

## Conclusions

Three different bulk GaAsBi samples regarding Bi distribution modes were investigated in this study using STEM techniques. The quantification of scattering cross sections was applied to a GaAs-GaAsBi hetero-diode grown on conventional (001) GaAs, showing atomically abrupt interface and early CuPt_B_-type ordering onset. Numerical multislice image simulations within the frozen-phonon thermal scattering approximation were used to investigate GaAsBi HAADF images. It showed that due to channeling, the configurational Bi variations can translate into apparent compositional variations. To carry out column-by-column Bi atom counting would thus require numerical image analysis. EDX mapping was presented of a single-variant ordered dilute GaAsBi sample grown on an offcut substrate. To avoid the configurational errors in elemental EDX quantification, the X-ray signals were averaged over many columns in (111) atomic planes, and the order parameter was estimated to be *η* = 0.07 in this sample. The atomic-resolution HAADF and EDX were also used to analyze a VCM GaAsBi film synthesized using a slow substrate rotation rate. This sample showed Bi content modulation in the [001] axis with a period of three lattice constants in addition to the CuPt_B_ ordering. Finally, bulk plasmon energy mapping using monochromated EELS was performed on a GaAs-GaAsBi hetero-diode. As the plasmon energy shift in dilute GaAsBi is related to the unit-cell volume changes, this provides a simple method to complement XRD-based techniques to examine local strain-state in GaAsBi alloys.

## Methods

Three different samples were examined in this study, samples S1, S2, and S3, all grown by solid-source MBE. The first sample, S1, is a GaAsBi *p-i-n* heterojunction, with an intrinsic 420-nm GaAsBi layer containing ~ 4.5 Bi%, as evaluated by XRD (not shown here) and room-temperature PL (SI Fig. S1). The n-type and p-type GaAs layers are 100 nm and 80 nm thick, respectively, and were doped to 5 × 10^17^ cm^−3^ concentrations using Si and Be, respectively. The sample was grown on an n-type (001) GaAs substrate using SVT-A MBE reactor equipped with metallic Ga and Bi sources and a two-zone valved arsenic cracker. The GaAs layers were deposited using a 330-nm/h growth rate at 600 ^°^C substrate temperature, supplying arsenic overpressure. GaAsBi layer was grown using a 100 nm/h rate, 10 revolutions per minute (RPM) substrate rotation, 360 ^°^C (thermocouple readings), As/Ga BEP around 1.08, Bi flux ~ 10^−7^ Torr. The (2 × 1) surface reconstructions were seen using RHEED during GaAsBi deposition. The second sample, S2, consisted of 280 nm thick GaAsBi with 1.0 eV band gap and~ 5.8 Bi%, as measured by PL (SI Fig. S1) and XRD [34]. This sample was grown over a ~ 300 nm GaAs buffer layer which was deposited on a *p*-type (001) Ge substrate with 6° offcut towards <110>. The first 50 nm of the buffer was deposited by migration-enhanced epitaxy. The remaining 300 nm of GaAs buffer was synthesized at 600 °C. GaAsBi film was grown at 350 °C, with BEP ratio of As to Ga in the range 1.063 to 1.1, and Bi/Ga ratio 0.35–0.37. The substrate was rotated 15 at RPM. The third and final sample, S3, was grown using the Veeco GENxplor MBE chamber, with the same type of sources and the arsenic cracker as in the SVT-A reactor. The sample consists of 500 nm GaAsBi with ~ 2.8% Bi, as measured by PL (SI Fig. 1) and XRD (not shown here). The layer was grown at 310 °C (band edge absorption measurement, kSA Bandit), on top of 80 nm GaAs buffer layer grown at 580 °C. The growth rate of the bismide was 0.5 monolayers/s, As/Ga BEP ratio ~ 1.35, and Bi flux ~ 8 × 10-8 Torr. The substrate was rotated at 5 RPM.

Transmission electron microscopy samples were prepared in a cross-sectional geometry by the focused ion beam (FIB) lift-out technique using FEI Helios Nanolab 650 dual-beam microscope. The samples were polished to 20–25 nm thickness, as measured by the EELS Log-ratio method, and argon-oxygen plasma-cleaned or degassed before loading into a microscope. HAADF imaging was carried out using cold-field emission double aberration-corrected JEOL JEM-ARM200CF operated at 200 kV [67]. The inner collection semi-angle of the HAADF detector was set to 90 mrad, with 22 mrad probe convergence semi-angle. The HAADF image analysis was carried out using StatSTEM add-on for Matlab [44]. Single width 2D Gaussian functions were fitted to the atomic columns after background subtraction. HAADF image simulation was performed using the muSTEM software using 15 frozen-phonon configurations, transmission functions with 0.02 Å square pixel size, and supercell size ~ 20 × 15 Å [68, 69]. The above experimental STEM probe parameters were used with defocus C1 = 0, C3 = 0.002 mm, and C5 = 1 mm spherical aberration coefficients, and a fully coherent electron probe. Kirkland multislice code was used to calculate the average of electron probe intensity versus sample depth, averaged over 10 frozen-phonon configurations [68]. The intensity average is taken across the atomic column in a 1 Å wide window. X-ray energy dispersive spectroscopy was performed using 0.98 steradian solid-angle windowless silicon drift-detector JEOL JED-2300. The probe current was set to 200 pA for EDX characterization and pixel dwell time 0.2 msec. The EDX images were 512 × 512 pixels in size, and a total of 5 frames were accumulated for each data set. Wiener filtering was applied to both EDX images for visualization, and sample drift-correction was used on Fig. 5 EDX data. On-axis electron energy-loss spectrum imaging was carried out using a modified monochromated Nion Hermes-200 (ChromaTEM) operated at 100 kV. The probe convergence semi-angle was set to 10 mrad, EELS collection semi-angle 35 mrad, 0.02 eV EELS energy dispersion, and 0.005 s EELS exposure time. The FWHM of the ZLP with beam positioned on the sample was measured to be 0.11 eV. Gatan DM 3.01 image analysis software was employed post-acquisition to center and removes the ZLP. The spectrum image was binned vertically by a factor of 4 and fully binned in the horizontal direction. Cross-correlation-based “Align SI by peak” algorithm was employed within the Gatan DM 3.01 software to determine plasmon peak shifts. Room-temperature PL measurements were carried out using a 420-mm focal length monochromator along with thermoelectrically cooled InGaAs photodetector. Diode-pumped solid-state laser emitting at the wavelength of 532 nm with an estimated power density of 5 kW/cm^2^ was used as an excitation source.

## Supplementary information

**Additional file 1: Figure S1.** Room-temperature photoluminescence spectra of GaAsBi samples presented in the main text. **Figure S2.** (a) The SCS histogram of the bottom GaAs buffer layer in Fig. 1 (a) (and also Fig. 2(a)) fitted with six Gaussians. The inset on the top right shows the quantified region. (b) Zoomed-in GaAs buffer layer with atomic columns indicated by squares according to the color-scheme in Fig. S2 (a). **Figure S3.** Shows the average probe intensity in a 1 Å wide window as a function of propagation depth in <110> GaAs crystal. The electron probe is positioned directly atop As column. The propagation simulation was averaged over 10 frozen-phonon configurations. Interference-based intensity oscillations can be seen as well as an overall intensity decay. see Methods for more details. **Figure S4.** EELS data showing a representative spectrum of GaAs (red) and GaAsBi (black) plasmon peaks. Zero-loss peaks have been centred and removed. The spectra are taken from the same data set as presented in Figure 3 (sample S1) and spatially binned, as detailed in the Methods section. **Figure S5.** Wiener filtered EDX elemental images from sample S3 that were used in the color-overlaid image Fig. 5 (b).

## Data Availability

The datasets used and/or analyzed during the current study are available from the corresponding author on reasonable request.

## Abbreviations

BEP: Beam equivalent pressure ratio
EELS: Electron energy-loss spectroscopy
FFT: Fast Fourier transform
HAADF: High-angle annular dark-field
MBE: Molecular beam epitaxy
PL: Photoluminescence
STEM: Scanning transmission electron microscopy
SCS: Scattering cross-section
VCM: Vertical composition modulations
EDX: X-ray energy dispersive spectroscopy

## References

[1] Batool Zahida, Chatterjee Sangam, Chernikov Alexej, Duzik Adam, Fritz Rafael, Gogineni Chaturvedi, Hild Konstanze, Hosea Thomas J.C., Imhof Sebastian, Johnson Shane R., Jiang Zenan, Jin Shirong, Koch Martin, Koch Stephan W., Kolata Kolja, Lewis Ryan B., Lu Xianfeng, Masnadi-Shirazi Mostafa, Millunchick Joanna Mirecki, Mooney Patricia M., Riordan Nathaniel A., Rubel Oleg, Sweeney Stephen J., Thomas John C., Thränhardt Angela, Tiedje Thomas, Volz Kerstin (2013). Bismuth-containing III–V semiconductors. Molecular Beam Epitaxy.

[2] Alberi K, Dubon OD, Walukiewicz W (2007). Valence band anticrossing in GaBi xAs 1-x. Appl Phys Lett.

[3] Deng HX, Li J, Li SS (2010). Band crossing in isovalent semiconductor alloys with large size mismatch: first-principles calculations of the electronic structure of Bi and N incorporated GaAs. Phys Rev B - Condens Matter Mater Phys.

[4] Fluegel B, Francoeur S, Mascarenhas A (2006). Giant spin-orbit bowing in GaAs1-xBix. Phys Rev Lett.

[5] Marko Igor P., Sweeney Stephen J. (2017). Progress Toward III–V Bismide Alloys for Near- and Midinfrared Laser Diodes. IEEE Journal of Selected Topics in Quantum Electronics.

[6] Richards RD, Mellor A, Harun F (2017). Photovoltaic characterisation of GaAsBi/GaAs multiple quantum well devices. Sol Energy Mater Sol Cells.

[7] Thomas T, Mellor A, Hylton N P, Führer M, Alonso-Álvarez D, Braun A, Ekins-Daukes N J, David J P R, Sweeney S J (2015). Requirements for a GaAsBi 1 eV sub-cell in a GaAs-based multi-junction solar cell. Semiconductor Science and Technology.

[8] Ptak AJ, France R, Beaton DA (2012). Kinetically limited growth of GaAsBi by molecular-beam epitaxy. J Cryst Growth.

[9] Kim Tong-Ho, Forghani Kamran, Collar Kristen, Kuech Thomas F., Brown April S. (2014). Growth of GaAs1−xBix by molecular beam epitaxy: Trade-offs in optical and structural characteristics. Journal of Applied Physics.

[10] Beaton D. A., Mascarenhas A., Alberi K. (2015). Insight into the epitaxial growth of high optical quality GaAs1–xBix. Journal of Applied Physics.

[11] Wood A, Wood AW, Chen W (2017). Annealing-induced precipitate formation behavior in MOVPE-grown GaAs1-xBi x explored by atom probe tomography and HAADF-STEM. Nanotechnology.

[12] Steele JA, Lewis RA, Horvat J, et al (2016) Surface effects of vapour-liquid-solid driven Bi surface droplets formed during molecular-beam-epitaxy of GaAsBi. Sci Rep 6:. 10.1038/srep28860 10.1038/srep28860 PMC4932629 27377213

[13] Norman Andrew G., France Ryan, Ptak Aaron J. (2011). Atomic ordering and phase separation in MBE GaAs1−xBix. Journal of Vacuum Science & Technology B, Nanotechnology and Microelectronics: Materials, Processing, Measurement, and Phenomena.

[14] Butkutė R, Niaura G, Pozingytė E, et al (2017) Bismuth quantum dots in annealed GaAsBi/AlAs quantum wells. Nanoscale Res Lett 12. 10.1186/s11671-017-2205-7 10.1186/s11671-017-2205-7 PMC5493604 28673054

[15] Marko I P, Jin S R, Hild K, Batool Z, Bushell Z L, Ludewig P, Stolz W, Volz K, Butkutė R, Pačebutas V, Geizutis A, Krotkus A, Sweeney S J (2015). Properties of hybrid MOVPE/MBE grown GaAsBi/GaAs based near-infrared emitting quantum well lasers. Semiconductor Science and Technology.

[16] Forghani K, Guan Y, Wood AW (2014). Self-limiting growth when using trimethyl bismuth (TMBi) in the metal-organic vapor phase epitaxy (MOVPE) of GaAs1-yBiy. J Cryst Growth.

[17] Beyer A, Knaub N, Rosenow P (2017). Local Bi ordering in MOVPE grown Ga(As,Bi) investigated by high resolution scanning transmission electron microscopy. Appl Mater Today.

[18] Gelczuk Ł, Kopaczek J, Rockett TBO (2017). Deep-level defects in n-type GaAsBi alloys grown by molecular beam epitaxy at low temperature and their influence on optical properties. Sci Rep.

[19] Mooney PM, Watkins KP, Jiang Z (2013). Deep level defects in n-type GaAsBi and GaAs grown at low temperatures. J Appl Phys.

[20] Joshya RS, Ptak AJ, France R (2014). Resonant state due to Bi in the dilute bismide alloy GaAs1-xBix. Phys Rev B - Condens Matter Mater Phys.

[21] Alberi K, Beaton DA, Mascarenhas A (2015) Direct observation of the E- resonant state in GaA s1-x B IX. Phys Rev B - Condens Matter Mater Phys 92:241201(R). 10.1103/PhysRevB.92.241201

[22] Bannow LC, Rubel O, Badescu SC (2016). Configuration dependence of band-gap narrowing and localization in dilute GaAs1-xBix alloys. Phys Rev B.

[23] Luo G, Yang S, Jenness GR (2017). Understanding and reducing deleterious defects in the metastable alloy GaAsBi. NPG Asia Mater.

[24] Ciatto G, Alippi P, Amore Bonapasta A, Tiedje T (2011). How much room for BiGa heteroantisites in GaAs1-xBix?. Appl Phys Lett.

[25] Wilson T, Hylton NP, Harada Y (2018). Assessing the nature of the distribution of localised states in bulk GaAsBi. Sci Rep.

[26] Imhof S, Thränhardt A, Chernikov A (2010). Clustering effects in Ga(AsBi). Appl Phys Lett.

[27] Mascarenhas A (2002) Spontaneous ordering in semiconductor alloys. Springer US

[28] Reyes DF, Bastiman F, Hunter CJ (2014). Bismuth incorporation and the role of ordering in GaAsBi/GaAs structures. Nanoscale Res Lett.

[29] Zunger A (1997). Spontaneous atomic ordering in semiconductor alloys: causes, carriers, and consequences. MRS Bull.

[30] Coll C., Barrigón E., López-Conesa L., Rebled J., Barrutia L., Rey-Stolle I., Estradé S., Algora C., Peiró F. (2017). The effect of Sb-surfactant on GaInP CuPtB type ordering: assessment through dark field TEM and aberration corrected HAADF imaging. Physical Chemistry Chemical Physics.

[31] Zhang SB, Froyen S, Zunger A (1995). Surface dimerization induced CuPtB versus CuPtA ordering of GaInP alloys. Appl Phys Lett.

[32] Occena J, Jen T, Lu H (2018). Surfactant-induced chemical ordering of GaAsN:Bi. Appl Phys Lett.

[33] Bastiman F, Cullis AG, David JPR, Sweeney SJ (2012). Bi incorporation in GaAs(100)-2 × 1 and 4 × 3 reconstructions investigated by RHEED and STM. J Cryst Growth.

[34] Paulauskas T, Pačebutas V, Geižutis A et al (2020) GaAs1-xBix growth on Ge: anti-phase domains, ordering, and exciton localization. Sci Rep 10. 10.1038/s41598-020-58812-y 10.1038/s41598-020-58812-y PMC7005183 32029827

[35] Wei SH, Zunger A (1994). Optical properties of zinc-blende semiconductor alloys: Effects of epitaxial strain and atomic ordering. Phys Rev B.

[36] Zhang Y, Mascarenhas A, Ernst P (1997). Effects of strain, substrate misorientation, and excitonic transition on the optical polarization of ordered zinc-blende semiconductor alloys. J Appl Phys.

[37] France RM, McMahon WE, Kang J (2014). In situ measurement of CuPt alloy ordering using strain anisotropy. J Appl Phys.

[38] Wei SH, Zunger A (1994). Optical anisotropy and spin polarization in ordered GaInP. Appl Phys Lett.

[39] Wei SH, Zunger A (1998). Fingerprints of CuPt ordering in III-V semiconductor alloys: Valence-band splittings, band-gap reduction, and x-ray structure factors. Phys Rev B - Condens Matter Mater Phys.

[40] Sales D. L., Guerrero E., Rodrigo J. F., Galindo P. L., Yáñez A., Shafi M., Khatab A., Mari R. H., Henini M., Novikov S., Chisholm M. F., Molina S. I. (2011). Distribution of bismuth atoms in epitaxial GaAsBi. Applied Physics Letters.

[41] Tait C. Ryan, Yan Lifan, Millunchick Joanna M. (2017). Droplet induced compositional inhomogeneities in GaAsBi. Applied Physics Letters.

[42] Skapas M, Stanionytė S, Paulauskas T, Butkutė R (2019). HRTEM Study of size-controlled Bi quantum dots in annealed GaAsBi/AlAs multiple quantum well Structure. Phys Status Solidi.

[43] Norman AG, France RM, McMahon WE (2013). The influence of atomic ordering on strain relaxation during the growth of metamorphic solar cells. J Phys Conf Ser.

[44] De Backer A, van den Bos KHW, Van den Broek W (2016). StatSTEM: an efficient approach for accurate and precise model-based quantification of atomic resolution electron microscopy images. Ultramicroscopy.

[45] Martinez GT, Rosenauer A, De Backer A (2014). Quantitative composition determination at the atomic level using model-based high-angle annular dark field scanning transmission electron microscopy. Ultramicroscopy.

[46] Findlay S.D., Allen L.J., Oxley M.P., Rossouw C.J. (2003). Lattice-resolution contrast from a focused coherent electron probe. Part II. Ultramicroscopy.

[47] Paulauskas T, Klie RFRF (2019). Decay of high-energy electron bound states in crystals. Ultramicroscopy.

[48] Allen LJ, Findlay SD, Oxley MP et al (2006) Channeling effects in high-angular-resolution electron spectroscopy. Phys Rev B - Condens Matter Mater Phys 73. 10.1103/PhysRevB.73.094104

[49] Geuens P., Dyck D. Van (2005). The S‐State Model for Electron Channeling in High‐Resolution Electron Microscopy. Advances in Imaging and Electron Physics Volume 136.

[50] Chen Z, Taplin DJ, Weyland M (2017). Composition measurement in substitutionally disordered materials by atomic resolution energy dispersive X-ray spectroscopy in scanning transmission electron microscopy. Ultramicroscopy.

[51] Chen Z, Weyland M, Sang X (2016). Quantitative atomic resolution elemental mapping via absolute-scale energy dispersive X-ray spectroscopy. Ultramicroscopy.

[52] MacArthur KE, Brown HG, Findlay SD, Allen LJ (2017). Probing the effect of electron channelling on atomic resolution energy dispersive X-ray quantification. Ultramicroscopy.

[53] Pryor A, Ophus C, Miao J (2017). A streaming multi-GPU implementation of image simulation algorithms for scanning transmission electron microscopy. Adv Struct Chem Imaging.

[54] Brown HG, Ciston J, Ophus C (2019). Linear-scaling algorithm for rapid computation of inelastic transitions in the presence of multiple electron scattering. Phys Rev Res.

[55] Dröge H, Fleszar A, Hanke W (1999). Complex loss function of CdTe. Phys Rev B - Condens Matter Mater Phys.

[56] Electron Energy-Loss Spectroscopy in the Electron Microscope, R.F. Egerton, Springer. https://www.springer.com/gp/book/9781441995827.

[57] Beanland R, Gass MH, Papworth AJ, et al (2005) Mapping quantum dot-in-well structures on the nanoscale using the plasmon peak in electron energy loss spectra. 1–8. 10.1103/PhysRevB.72.075339

[58] Palisaitis J, Hsiao CL, Junaid M et al (2011) Effect of strain on low-loss electron energy loss spectra of group-III nitrides. Phys Rev B - Condens Matter Mater Phys 84. 10.1103/PhysRevB.84.245301

[59] France R., Jiang C.-S., Ptak A. J. (2011). In situ strain relaxation comparison between GaAsBi and GaInAs grown by molecular-beam epitaxy. Applied Physics Letters.

[60] Sieg RM, Ringel SA, Ting SM (1998). Anti-phase domain-free growth of GaAs on offcut (001) Ge wafers by molecular beam epitaxy with suppressed Ge outdiffusion. J Electron Mater.

[61] Hudait MK, Krupanidhi SB (2001). Self-annihilation of antiphase boundaries in GaAs epilayers on Ge substrates grown by metal-organic vapor-phase epitaxy. J Appl Phys.

[62] Wang P, Pan W, Wu X (2016). Heteroepitaxy growth of GaAsBi on Ge(100) substrate by gas source molecular beam epitaxy. Appl Phys Express.

[63] Phillips Patrick J., Paulauskas Tadas, Rowlands Neil, Nicholls Alan W., Low Ke-Bin, Bhadare Santokh, Klie Robert F. (2014). A New Silicon Drift Detector for High Spatial Resolution STEM-XEDS: Performance and Applications. Microscopy and Microanalysis.

[64] Paulauskas T, Sen FG, Sun C (2019). Stabilization of a monolayer tellurene phase at CdTe interfaces. Nanoscale.

[65] He Xiaoqing, Paulauskas Tadas, Ercius Peter, Varley Joel, Bailey Jeff, Zapalac Geordie, Poplavskyy Dmitry, Mackie Neil, Bayman Atiye, Spaulding David, Klie Robert, Lordi Vincenzo, Rockett Angus (2017). Cd doping at PVD-CdS/CuInGaSe2 heterojunctions. Solar Energy Materials and Solar Cells.

[66] Stevens MA, Grossklaus KA, McElearney JH, Vandervelde TE (2019). Impact of rotation rate on bismuth saturation in GaAsBi grown by molecular beam epitaxy. J Electron Mater.

[67] Klie R. F., Gulec A., Guo Z., Paulauskas T., Qiao Q., Tao R., Wang C., Low K. B., Nicholls A. W., Phillips P. J. (2013). The new JEOL JEM-ARM200CF at the University of Illinois at Chicago. Crystal Research and Technology.

[68] Kirkland Earl J. (2010). Advanced Computing in Electron Microscopy.

[69] Allen LJ, D’Alfonso AJ, Findlay SD (2015). Modelling the inelastic scattering of fast electrons. Ultramicroscopy.

