# Supplementary material for: Atomic-Resolution EDX, HAADF, and EELS Study of GaAs1-xBix Alloys
Source: Nanoscale Res Lett. 2020 May 25;15:121. doi: 10.1186/s11671-020-03349-2 (PMC7248167; doi:10.1186/s11671-020-03349-2)

**Supplementary Information**

Tadas Paulauskas,^1*^ Vaidas Pačebutas,^1^ Renata Butkutė,^1^ Bronislovas Čechavičius,^1^ Arnas Naujokaitis,^1^ Mindaugas Kamarauskas,^1^ Martynas Skapas,^1^ Jan Devenson,^1^ Mária Čaplovičová,^2^ Viliam Vretenár,^2^ Xiaoyan Li,^3^ Mathieu Kociak^3^ and Arūnas Krotkus^1^

^1^Center for Physical Sciences and Technology, Saulėtekio al. 3, Vilnius, Lithuania

^2^STU Centre for Nanodiagnostics, University Science Park Bratislava Centre, Slovak University of Technology, Vazovova 5, Bratislava, Slovakia

^3^Solid State Physics Laboratory, University of Paris SUD, 91400 Orsay, France

*tadas.paulauskas@ftmc.lt

**Figure S1**. Room-temperature photoluminescence spectra of GaAsBi samples presented in the main text.


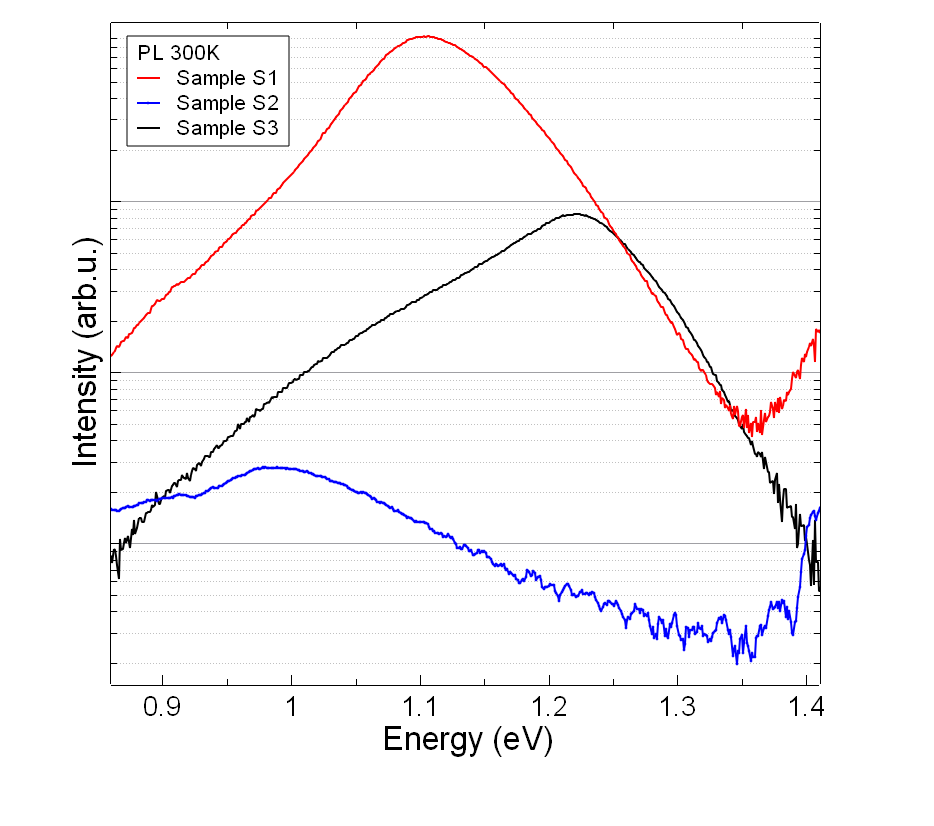


**Figure S2**. (a) The SCS histogram of the bottom GaAs buffer layer in Fig. 1 (a) (and also Fig. 2(a)) fitted with six Gaussians. The inset on the top right shows the quantified region. (b) Zoomed-in GaAs buffer layer with atomic columns indicated by squares according to the color-scheme in Fig. S2 (a).


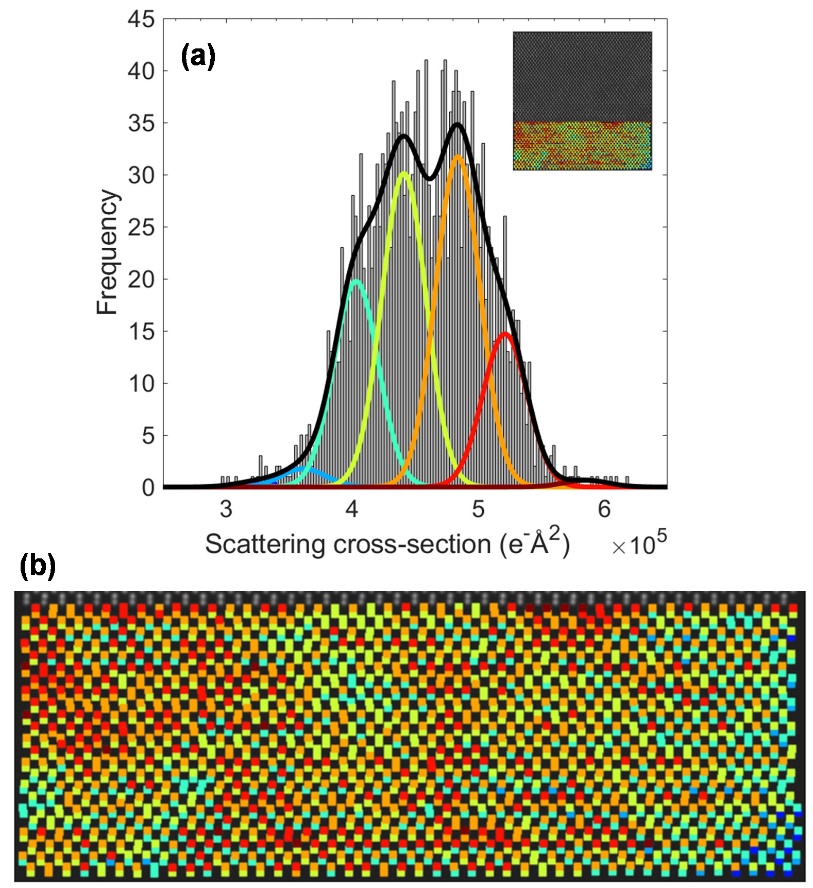

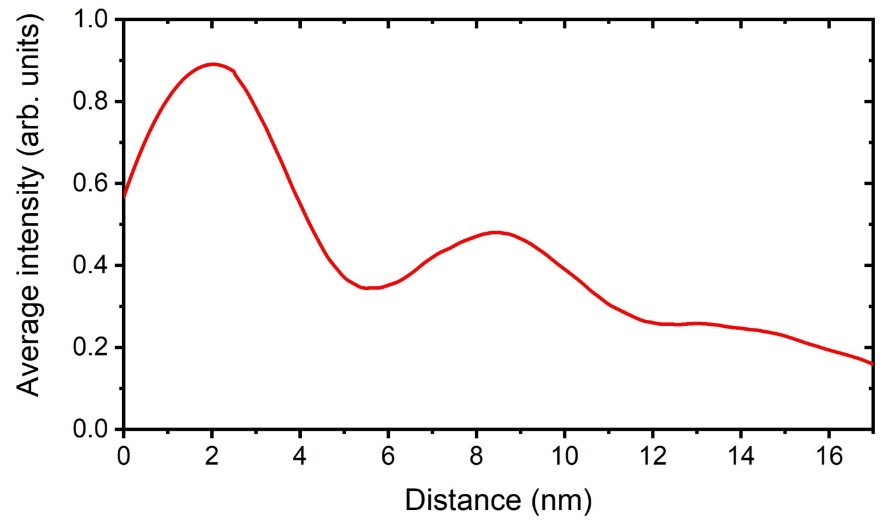


**Figure S3**. Shows the average probe intensity in a 1 Å wide window as a function of propagation depth in <110> GaAs crystal. The electron probe is positioned directly atop a As column. The propagation simulation was averaged over 10 frozen-phonon configurations. Interference-based intensity oscillations can be seen as well as an overall intensity decay. See Methods for more details.

**Figure S4**. EELS data showing a representative spectrum of GaAs (red) and GaAsBi (black) plasmon peaks. Zero-loss peaks have been centred and removed. The spectra are taken from the same data set as presented in Figure 3 (sample S1) and spatially binned, as detailed in the Methods section.


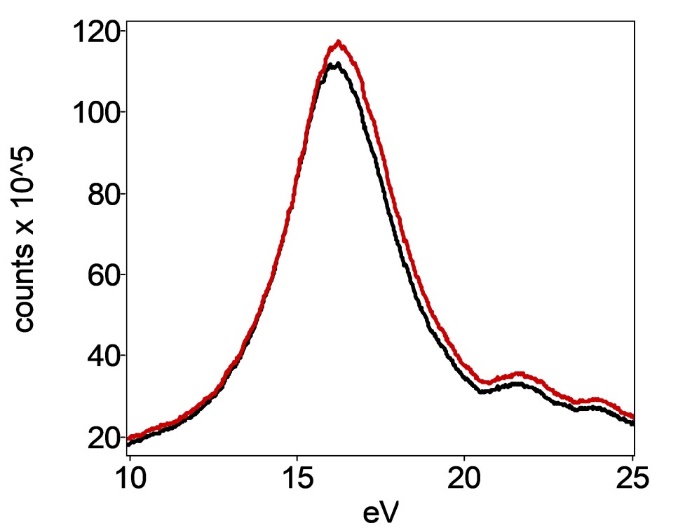


**Figure S5**. Wiener filtered EDX elemental images from sample S3 that were used in the color-overlaid image Fig. 5 (b).


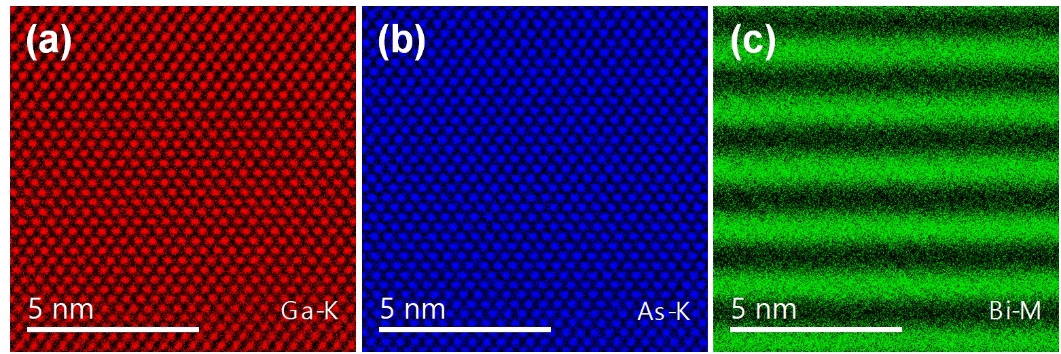

Supplement: Supplementary file 1 — Additional file 1: Figure S1. Room-temperature photoluminescence spectra of GaAsBi samples presented in the main text. Figure S2. (a) The SCS histogram of the bottom GaAs buffer layer in Fig. 1 (a) (and also Fig. 2(a)) fitted with six Gaussians. The inset on the top right shows the quantified region. (b) Zoomed-in GaAs buffer layer with atomic columns indicated by squares according to the color-scheme in Fig. S2 (a). Figure S3. Shows the average probe intensity in a 1 Å wide window as a function of propagation depth in <110> GaAs crystal. The electron probe is positioned directly atop As column. The propagation simulation was averaged over 10 frozen-phonon configurations. Interference-based intensity oscillations can be seen as well as an overall intensity decay. see Methods for more details. Figure S4. EELS data showing a representative spectrum of GaAs (red) and GaAsBi (black) plasmon peaks. Zero-loss peaks have been centred and removed. The spectra are taken from the same data set as presented in Figure 3 (sample S1) and spatially binned, as detailed in the Methods section. Figure S5. Wiener filtered EDX elemental images from sample S3 that were used in the color-overlaid image Fig. 5 (b). [file 11671_2020_3349_MOESM1_ESM.docx]
